# Use of a high platelet-to-RBC ratio of 2:1 is more effective in correcting trauma-induced coagulopathy than a ratio of 1:1 in a rat multiple trauma transfusion model

**DOI:** 10.1186/s40635-019-0242-5

**Published:** 2019-07-25

**Authors:** Derek J. B. Kleinveld, Mathijs R. Wirtz, Daan P. van den Brink, M. Adrie W. Maas, Joris J. T. H. Roelofs, J. Carel Goslings, Markus W. Hollmann, Nicole P. Juffermans

**Affiliations:** 1 Department of Intensive Care Medicine, Amsterdam UMC, Meibergdreef 9, 1105 AZ Amsterdam, The Netherlands; 2 Laboratory of Experimental Intensive Care and Anesthesiology, Amsterdam UMC, Amsterdam, The Netherlands; 3 Department of Trauma Surgery, Amsterdam UMC, Amsterdam, The Netherlands; 4 Department of Pathology, Amsterdam UMC, Amsterdam, The Netherlands; 5 Department of Trauma Surgery, Onze Lieve Vrouwe Gasthuis, Amsterdam, The Netherlands; 6 Department of Anesthesiology, Amsterdam UMC, Amsterdam, The Netherlands

**Keywords:** Trauma, Transfusion, Coagulopathy, Experimental, Platelets

## Abstract

**Background:**

Platelet dysfunction importantly contributes to trauma-induced coagulopathy (TIC). Our aim was to examine the impact of transfusing platelets (PLTs) in a 2:1 PLT-to-red blood cell (RBC) ratio versus the standard 1:1 ratio on transfusion requirements, correction of TIC, and organ damage in a rat multiple trauma transfusion model.

**Methods:**

Mechanically ventilated male Sprague Dawley rats were traumatized by crush injury to the small intestine and liver and a fracture of the femur, followed by exsanguination until a mean arterial pressure (MAP) of 40 mmHg. Animals were randomly assigned to receive resuscitation in a high PLT dose (PLT to plasma to RBC in a ratio of 2:1:1) or a standard PLT dose (ratio of 1:1:1) until a MAP of 60 mmHg was reached (*n* = 8 per group). Blood samples were taken for biochemical and thromboelastometry (ROTEM) assessment. Organs were harvested for histopathology.Outcome measures were transfusion requirements needed to reach a pretargeted MAP, as well as ROTEM correction and organ failure.

**Results:**

Trauma resulted in coagulopathy as assessed by deranged ROTEM results. Mortality rate was 19%, with all deaths occurring in the standard dose group. The severity of hypovolemic shock as assessed by lactate and base excess was not different in both groups. The volume of transfusion needed to reach the MAP target was lower in the high PLT dose group compared to the standard dose, albeit not statistically significant (*p* = 0.054). Transfusion with a high PLT dose resulted in significant stronger clot firmness compared to the standard dose at all time points following trauma, while platelet counts were similar. Organ failure as assessed by biochemical analysis and histopathology was not different between groups, nor were there any thromboembolic events recorded.

**Conclusions:**

Resuscitation with a high (2:1) PLT-to-RBC ratio was more effective compared to standard (1:1) PLT-to-RBC ratio in treating TIC, with a trend towards reduced transfusion volumes. Also, high PLT dose did not aggravate organ damage. Transfusion strategies using higher PLT dose regiments might be a feasible treatment option in hemorrhaging trauma patients for the correction of TIC.

## Background

Traumatic injury initiates a variety of coagulation processes, often resulting in trauma-induced coagulopathy (TIC) [1, 2]. TIC occurs in approximately one third of trauma patients and contributes to early mortality by exsanguination [3, 4], but is also associated with the development of multiple organ dysfunction syndrome (MODS) [5–7], thereby increasing the length of stay in the intensive care [8].

Historically, resuscitation of traumatic bleeding had focused on volume replacement therapy using mainly red blood cell (RBC) products, while TIC was generally ignored [9, 10]. The PROPPR trial showed that a balanced resuscitation approach using platelets (PLT), plasma, and RBC in a 1:1:1 ratio was more effective in achieving hemostasis than a lower 1:1:2 ratio [11]. These findings were confirmed in observational studies in both military and civilian trauma cohorts, suggesting an improved survival when higher PLT-to-RBC ratios (1:1) were used [12, 13].

Although platelet counts are typically normal after trauma, evidence is accumulating that platelet aggregation and activation pathways can be severely impaired [14]. Furthermore, dysfunctional platelets are independently associated with mortality in trauma patients [15]. These findings indicate that an impaired platelet function may be one of the key elements driving TIC and subsequent adverse outcomes. Thereby, PLT transfusion may compensate for decreased platelet functionality, resulting in treatment of TIC. Although current resuscitation strategies aim to reach a 1:1 PLT-to-RBC ratio, it is unknown whether increasing the dose to even higher ratios (> 1:1) may further improve clot formation, resulting in earlier hemostasis with subsequent improved outcome. On the other hand, high PLT doses may also predispose patients to the development of adverse events, mediated by platelet aggregation, thereby promoting (micro) thrombi formation, which may lead to thromboembolic events and/or MODS [16]. A possible mechanism of harm leading to these adverse events might be the release of trauma alarmin high-mobility group box 1 (HMGB-1), passively released by endothelial cell damage and actively secreted by platelets [17, 18]. In trauma, HMGB-1 is increased and was found to be a key player in thrombus formation in mice [17].

The purpose of this study was to investigate the risks and benefits of resuscitation by comparing a high PLT dose (2:1:1 PLT to plasma to RBC), with the current standard PLT dose (1:1:1 PLT to plasma to RBC) in an experimental trauma transfusion rat model.

## Methods

### Animals

Male Sprague Dawley (Envigo) rats (350–400 g) were used for all experiments. The study was approved by the animal care and use committee of the Amsterdam University Medical Centers, Netherlands. All procedures were performed in compliance with the Institutional Standards for Use of Laboratory Animals.

### Description of blood products preparation

Transfusion products were made from syngeneic donor rats by heart puncture and stored according to the national blood bank standards as described before [19, 20]. In short, plasma products were made by centrifugation of whole blood (10 min, 1892 g, 20 °C). Plasma was removed from the buffy coat, pooled from two donors, and stored at − 80 °C. Thawing of plasma occurred in ice water for 45–60 min on the day of the experiment. Rat PLT product was made using the buffy coat, which was diluted using pooled plasma, until a hematocrit of approximately 20%. After this, the platelet-rich plasma was centrifuged again (10 min, 288 g, 20 °C) to remove the remaining red blood cells and leucocytes. PLT products were stored under agitation on a roller bank in a culture flask for 1 day at 22 °C under a 5% CO_2_/95% air mixture. RBC products were made by diluting erythrocytes with saline-adenine-glucose-mannitol (SAGM, Fresenius Hemocare, Bad Homburg, Germany) to a hematocrit of 60% [20]. RBCs were stored at 4 °C for 1 day. Blood compatibility was addressed in previous experiments in which the blood from syngeneic rats was crossmatched without any signs of hemolysis [20].

### Description of trauma-transfusion model

A clinically relevant trauma-transfusion model was used [21] that was characterized by TIC, including platelet dysfunction [22, 23]. Rats were anesthetized with a mixture of ketamine (100 mg/ml, Eurovet), dexmedetomidine (0.5 mg/ml, Orion Pharma), and atropine (0.5 mg/ml, Centrafarm). A tracheostomy was performed after which animals were connected to a mechanical ventilator (Babylog 8000, Dräger) for pressure-controlled ventilation (10 cm H_2_0 peak inspiratory pressure, 5 cm H_2_0 positive end-expiratory pressure, FiO_2_ at 30%). Recruitment maneuvers were performed every 60 min by increasing peak inspiratory pressure to 30 cm H_2_0 for 5 breaths. The carotid artery was cannulated for arterial blood pressure monitoring and blood sampling, while the jugular vein was cannulated for the transfusion of blood products. Temperature was monitored continuously by a rectal thermometer.

Rats were traumatized by median laparotomy, crush injury to the small intestines and liver, and by fracture of a femur. Crush injury was induced by clamping of the intestine or liver for 3 s using a metal clamp covered with silicone tubing. The femur fracture was established using a blunt guillotine composing of 650 g of steel, which was dropped from a height of 14 cm on the right femur [21]. Then, animals were hemorrhaged until a mean arterial pressure (MAP) of 40 mmHg was reached (± 30% of circulating volume). Animals were randomized (*n* = 8 per group) to receive resuscitation with either 1:1:1 (standard PLT dose) or 2:1:1 (high PLT dose) PLT-to-plasma-to-RBC ratio at a rate of 8 ml/hour. Six hours after trauma, the rats were terminated by exsanguination, and the organs (lung, kidneys, liver, small intestine) were harvested for histopathological analysis.

### Outcomes

Primary outcome was the amount of transfusion product needed to reach a predefined MAP of 60 mmHg. Secondary outcomes were the correction of coagulation assays and organ failure assessment.

### Measurements

**Fig. 1:**
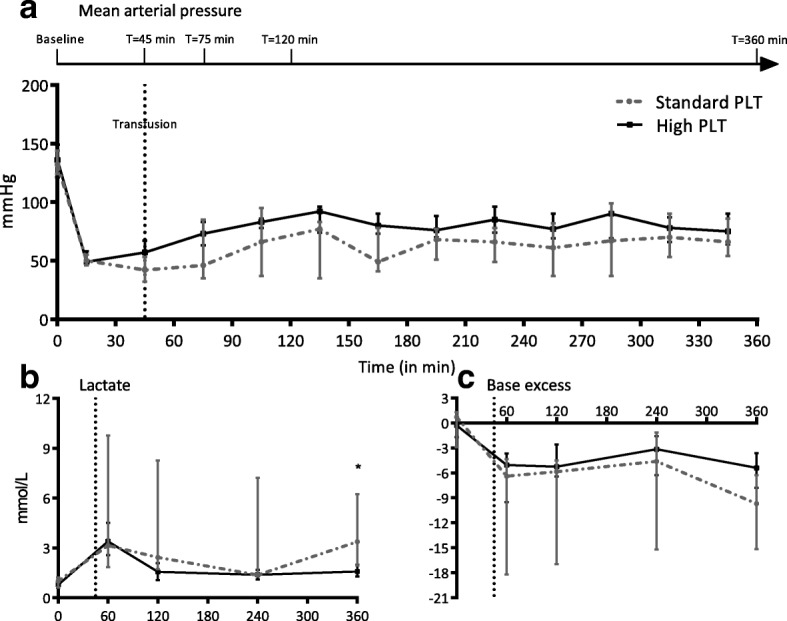
Shock parameters. Data are presented as median (IQR). **a** Mean arterial pressure. Line with arrow represents specific time points of coagulation status assessment during the experiment. Baseline (=before trauma), *T* = 45 min (=just before initiation of resuscitation), *T* = 75, *T* = 120, and T = 360 min (after injury). **b** Lactate. **c** Base excess. PLT platelet transfusion, MAP mean arterial pressure. Dotted vertical line at 45 min represents initiation of resuscitation. *p < 0.050 between groups

Blood samples were taken before trauma (*T* = 0), right before transfusion was initiated (*T* = 45 min) and at several time points after injury (*T* = 75, *T* = 120, *T* = 360 min) (Fig. 1). To determine adequate ventilation and assess shock parameters, blood gas analyses were performed at the same time points. Furthermore, blood samples were taken to assess platelet concentration and biochemical markers of organ injury, including aspartate aminotransferase (AST) and alanine transaminase (ALT), lactate dehydrogenase, and creatinine. Additionally, in urine samples, the amount of protein was determined.

Coagulation status of the rats was measured by rotational thromboelastometry (ROTEM Delta, Werfen, Spain). To obtain a functional profile of coagulation, the EXTEM assay was used to evaluate the extrinsic pathway using tissue factor to initiate coagulation. The FIBTEM assay, containing cytochalasin D (a potent platelet inhibitor), was used to evaluate the contribution of fibrinogen to clot formation. Analyses were performed according to the guideline supplied by the manufacturer. All assays were run for at least 60 min. Clotting amplitude at 5 min (CA5) and maximum clot firmness (MCF) were used to determine coagulopathy, as they are shown to be valid predictors for the assessment of TIC [24–26]. Evaluation platelet contribution to clot formation was assessed by subtracting FIBTEM MCF from EXTEM MCF assay (EXTEM-FIBTEM).

Enzyme-linked immunosorbent assays were performed to address interleukin-6 (DY506, R&D systems), interleukin-8 (DY525, R&D systems), and HMGB-1 (E-EL-R0505, ELabscience) according to instructions given by the manufacturer.

### Organ score

Hematoxylin & eosin (H&E) staining was performed for histopathological assessment. All organs were examined by a specialized pathologist, who was blinded to treatment allocation. Organs were scored on a scale of 0 to 3 (0 = absent, 1 = mild, 2 = moderate, 3 = severe) based on four categories. The scoring system was used earlier in our lab [21, 27] and was based on previous literature [28]. In short, lungs were scored based on edema, interstitial inflammation, endothelial inflammation, and hemorrhage. Kidneys were similarly scored the by presence of epithelial or luminal necrosis, tubular dilation, hemorrhage and neutrophil extravasation, and hemorrhage. Liver injury was assessed by scoring the degree of necrosis, hemorrhage, portal inflammation, and neutrophil infiltration. Lastly, the small intestines were scored based on swelling of villi, necrosis, hemorrhage, and neutrophil infiltration in the submucosa. The sum of the individual organ scores was calculated to determine the mean total organ scores.

### Statistical analysis

The sample size was based on an estimated difference in protein leakage in a previously done rat transfusion model with a mean difference of 8 ng/g lung homogenate and a standard deviation of 4 ng/g [20]. Using a two-sided *t* test (*α* = 0.05, *β* = 0.80), a total of six rats per group was needed.

Analyses were done using IBM SPSS Statistics version 24, and graphs were made using GraphPad Prism 7. After checking for normality using the Kolmogorov-Smirnov test and visual histogram examination, data were represented as median (IQR). Nonparametric samples were compared between groups with a Mann-Whitney *U* test. To test for differences in paired data, the nonparametric Wilcoxon signed-rank test was used. A *p* value of less than 0.05 was considered to be statistically significant.

## Results

### Effect of resuscitation strategy on hemodynamic variables

Baseline characteristics did not differ significantly between the standard and the high PLT dose group. Also, after the rats were traumatized and hemorrhaged, both groups had similar vital and biochemical parameters (Table 1). A decrease in MAP with an increase in lactate levels and decrease in base excess (BE) indicates the severity of hypovolemic shock (Fig. 1). The hemorrhaged volume to reach a MAP of 40 mmHg was similar between groups, with 21.6 [20.1–24.1] ml/kg in the standard PLT group vs 21.4 [20.1–24.9] ml/kg bodyweight in the high PLT group (*p* = 1.0). After resuscitation with high PLT dose, rats seemed to be hemodynamically more stable with a higher MAP throughout the experiment, albeit not reaching statistical significance. A trend towards a shorter resuscitation time to reach the predefined MAP of 60 mmHg was found in the high PLT dose group (19 [7–33] min) compared to the standard PLT dose group (54 [18–160] min, *p* = 0.054). Similarly, lower volumes of transfusion products were used to reach a MAP ≥60 mmHg (2.5 [0.9–4.3] vs 7.2 [2.4–10.0] ml) in the high platelet dose group, although not statistically significant (*p* = 0.054) (Table 2). Also, rats receiving the high PLT dose showed signs of an improved shock reversal, as lactate levels 6 h post injury were significantly lower in the high PLT dose group (1.6 [1.3–2.0] mmol/L) compared to the standard PLT dose group (3.4 [1.9–6.2] mmol/L, *p* = 0.013).

**Table 1:** Vital parameters at baseline and after induction of injury

Parameter	Standard PLT dose		High PLT dose	
	Before injury	After injury	Before injury	After injury
Weight (gram)	372 (364–384)	ND	388 (366–395)	ND
MAP (mmHg)	132 (124–144)	42 (32–53)	136 (121–149)	57 (43–67)
HR (bpm)	280 (260–300)	280 (208–308)	280 (273–288)	240 (220–260)
Temperature (°C)	36.6 (36.0–37.4)	37.0 (36.6–37.2)	36.3 (36.0–36.4)	36.8 (36.3–37.0)
pH	7.46 (7.35–7.50)	7.36 (7.22–7.39)	7.39 (7.36–7.42)	7.37 (7.31–7.40)
pCO2 (mmHg)	36.1 (30.7–39.4)	28.1 (19.1–36.5)	42.0 (39.5–43.4)	34.3 (31.4–36.8)
BE (mmol/L)	0.7 (−2.9–1.3)	− 6.4 (− 18.2 to − 4.3)	− 0.3 (− 1.7 to − 0.6)	− 5.1 (− 9.5 to − 3.8)
Lactate (mmol/L)	1.0 (0.6–1.2)	3.1 (1.9–9.8)	0.8 (0.6–1.0)	3.4 (2.6–4.5)
Hb (mmol/L)	8.5 (8.2–9.2)	6.8 (6.5–7.4)	9.7 (9.1–10.4)	7.6 (7.0–7.9)

Data are presented as median (IQR)

*PLT* platelet transfusion, *MAP* mean arterial pressure, *HR* heart rate, *bpm* beats per minute, *BE* base excess, *Hb* hemoglobin, *ND* not determined

**Table 2:** Transfusion

	Standard PLT dose	High PLT dose	*p* value
Hemorrhaged (ml)	8.0 (8.0–9.0)	8.0 (8.0–9.5)	0.870
Hemorrhaged per kg bodyweight (mL/kg)	21.6 (20.1–24.1)	21.4 (20.1–24.9)	1.000
Time until MAP > 60 mmHg was reached (min)	54 (18–160)	19 (7–33)	0.054
Transfusion needed to reach MAP > 60 mmHg (mL)	7.2 (2.4–10.0)	2.5 (1.0–4.3)	0.054
Total volume transfused (mL)	10 (10–10)	10 (10–10)	0.690

Data are presented as median (IQR)

*PLT* platelet transfusion, *MAP* mean arterial pressure

### Effect of resuscitation strategy on the correction of TIC

**Fig. 2:**
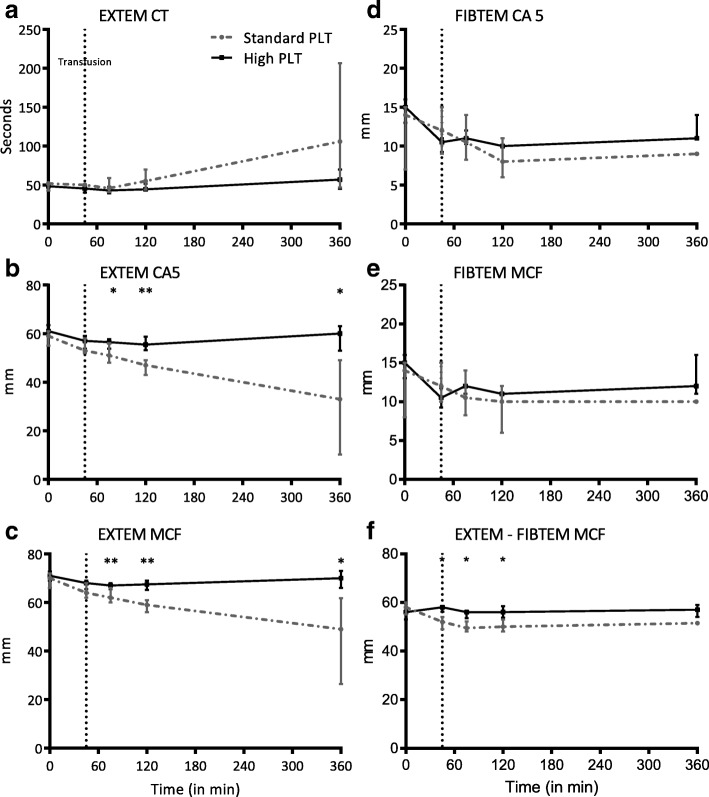
ROTEM assays. Data are presented as median (IQR). **a** EXTEM CT. **b** EXTEM CA5. **c** EXTEM MCF. **d** FIBTEM CA5. **e** FIBTEM MCF. **f** EXTEM - FIBTEM MCF. PLT platelet transfusion, CT clotting time, CA5 clotting amplitude at 5 min, MCF maximum clot firmness. Dotted vertical line at 45 min represents initiation of resuscitation. *p < 0.050, **p < 0.001 between groups

Trauma and hemorrhage resulted in severe derangements of the coagulation status of the rats. During the course of the experiment, the platelet count dropped in both groups (Table 3). The platelet count dropped significantly in the high PLT dose group (*p* = 0.040), but not in the low dose group. However, platelet counts did not differ between groups. Deranged ROTEM values showed an earlier correction in the high PLT dose group compared to the standard PLT dose group (Fig. 2). EXTEM CA5 and MCF were significantly lower in the standard PLT dose group after initiation of transfusion compared to the high PLT dose group. Maximum clot firmness (EXTEM MCF) in the standard dose group was lower than the high PLT dose group at 75 min (62 [60–66] vs 67 [66–68] mm, *p* = 0.006), at 120 min post injury (59 [56–61] vs 68 [65–69] mm, *p* < 0.001) and at 360 min post injury (49 [27–62] vs 70 [66–73] mm, *p* = 0.024), while FIBTEM MCF was equal in both groups at 75 min post injury (11 [8–14] vs 12 [11, 12] mm, *p* = 0.5), at 120 min post injury (10 [6–12] vs 11 [10–12] mm, *p* = 0.2) and at 360 min after injury (10 [10] vs 12 [11–16] mm, p = 0.2). Assessing platelet function by EXTEM-FIBTEM MCF resulted in a similar pattern; however, after initiation of transfusion, this parameter was already lower in the standard dose group compared to the high dose group (52 [49–54] vs 58 [56–59] mm, *p* = 0.014) (Fig. 2). Furthermore, fibrinogen levels after resuscitation were significantly lower in the standard PLT dose group compared to the high PLT dose group (Table 3).

**Table 3:** Organ injury and inflammation

Parameter	Standard PLT dose		High PLT dose	
	Before injury	After 6 h	Before injury	After 6 h
Respiratory				
PF-ratio (mmHg)	507 (487–561)	552 (372–621)	521 (478–553)	602 (517–620)
Coagulation				
Platelets (10^9^/L)*	877 (876–907)	419 (125–629)	1008 (946–1064)	675 (185–739)^#^
Fibrinogen (g/L)	1.5 (0.9–1.7)	1.3 (0.3–1.4)	1.6 (1.5–1.7)	1.7 (1.7–1.9)*
LDH (U/L)	129 (65–272)	519 (156–1179)	117 (80–211)	452 (261–1653)^#^
Liver				
AST (U/L)	63 (60–68)	277 (152–385)	66 (64–75)	332 (216–390)^#^
ALT (U/L)	55 (50–59)	168 (107–2613)^#^	57 (50–61)	146 (110–546)^#^
Kidney				
Creatinine (μmol/L)	25 (21–28)	103 (49–162)^#^	24 (22–26)	73 (42–136)^#^
Urine protein (g/L)	0.27 (0.08–0.55)	4.44 (2.40–11.09)	0.17 (0.10–0.33)	2.08 (1.68–3.24)^#^
Inflammation				
Leukocytes (10^9^/L)*	7.1 (4.4–7.7)	5.4 (5.2–6.6)	7.1 (4.4–7.7)	6.3 (4.9–7.7)
IL-6 (pg/mL)	< 4.1	< 4.1	< 4.1	< 4.1
IL-8 (pg/mL)	< 31.25	< 31.25	< 31.25	< 31.25
HMGB-1 (pg/mL)	402 (390–481)	689 (416–1877)^#^	380 (353–424)	659 (589–1276)^#^

Data are presented as median (IQR)

*PLT* platelet transfusion, *PF* PaO_2_/FiO_2_, *PT* prothrombin time, *AST* aspartate aminotransferase, *ALT* alanine transaminase, *LDH* lactate dehydrogenase, *IL-6* interleukin-6, *IL-8* interleukin-8, and *HMGB-1* high mobility group box 1

*p < 0.050 for differences between groups. ^#^*p* < 0.050 for differences before and after 6 h within group

### Effect of resuscitation therapy on parameters of organ injury and host inflammatory response

Biochemical assessment showed increased AST, ALT, and creatinine levels after injury; however, levels were not different between treatment groups (Table 3).

**Fig. 3:**
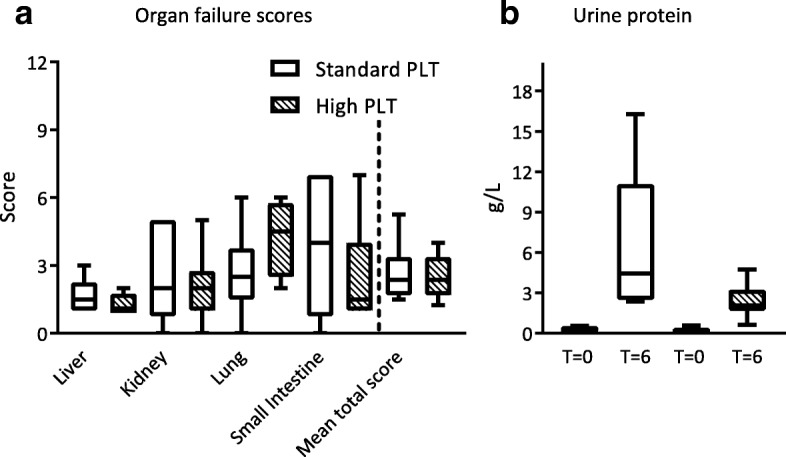
Organ failure. Data are presented as median (IQR). PLT platelet transfusion. Due to mortality, histopathological analysis was done on 6/7 rats in standard PLT dose group and 8/8 in high PLT dose group. **a** Organ failure scores. **b** Urine protein

The histology assessment was similar in both groups, indicating an equal amount of organ damage during the experiment in both groups (Table 2 and Fig. 3). Of note, (micro) thrombi formation in organs was absent in both groups. Interestingly, the small intestine was most severely injured in the standard PLT dose group, while the lungs were most damaged in the high PLT dose group.

Levels of HMGB-1 were significantly elevated after trauma compared to baseline in both groups. However, there was no significant difference between standard and high PLT dose groups (Table 2). In plasma, levels of interleukin-6 and interleukin-8 were below the detection limit.

### Mortality

Mortality rate in this experiment was 19%, with all deaths occurring in the standard PLT dose group. One rat died during induction of anesthesia and was excluded from all analyses. The remaining rats in the standard PLT dose group reached our primary outcome, therefore these rats were incorporated in our primary outcome analysis. Two rats died during the course of the experiment, of which one rat died 30 min prior to planned termination of the experiment. The last-mentioned animal was included in all analysis.

## Discussion

The results indicate that, in a rat model of multiple trauma and polytransfusion, a high dose PLT transfusion seems beneficial in correcting coagulopathy, possibly allowing for lower transfusion volumes compared to resuscitation with a standard PLT dose, while not aggravating organ damage.

In the high PLT dose group, the correction of TIC was associated with lower volumes of transfusion products needed to achieve a predefined hemodynamic goal. Although statistical significance was not reached, the earlier increase in MAP in the high PLT dose treated group was paralleled by a decrease in lactate levels and base excess, suggesting a more effective shock reversal. However, although the hemodynamic target of a MAP of 60 mmHg was reached with less transfusion volume in the high PLT dose group, the total amount of volume needed to maintain a MAP of 60 mmHg throughout the experiment was not different between groups. This may be due to the severity of the model, in which animals that are not resuscitated die [21]. Of note, two rats in the control group died after receiving the maximum amount (10 ml) of transfusion product, while no deaths occurred in the high PLT dose group. Taken together, these findings suggest improved hemostatic control in the high PLT dose group, resulting in earlier shock reversal.

This experimental trauma-transfusion model was characterized by deranged ROTEM parameters of clot firmness. Resuscitation with a high PLT dose significantly improved EXTEM CA5 and MCF compared to the standard PLT dose. Interestingly, fibrinogen concentration after resuscitation was higher in the high PLT dose group compared to the standard PLT dose group. A possible explanation may be that earlier hemostasis limited fibrinogen consumption. Alternatively, supplementation of fibrinogen by more platelet-rich plasma in the high PLT dose group may have resulted in higher fibrinogen levels. A recent study comparing fibrinogen concentration in different products showed similar fibrinogen levels in platelet-poor and platelet-rich plasma [29, 30], supporting the finding of more fibrinogen administration by transfusion of more plasma. However, given that FIBTEM CA5 and MCF were similar in both groups, it does not seem likely that higher levels of fibrinogen alone accounted for earlier shock reversal. Of note, in both groups, platelet counts decreased, which reached a statistical significance drop only in the animals receiving a high PLT dose. We do not have a clear explanation for this finding. Due to low numbers, this difference may have resulted by chance. Of importance, even with a significant drop in platelet count, ROTEM-derived parameters of coagulopathy improved in the high PLT group. The ROTEM results on EXTEM-FIBTEM MCF support the finding that platelet function is severely disturbed after trauma, and suggest that active platelets are needed to restore TIC [24].

Transfusion is associated with organ failure in trauma, in particular, lung injury, which seems to be a dose-dependent effect [31–33]. Also, a retrospective study in trauma patients suggests that high doses of PLT products are associated with lung injury [34], although this is not found in all studies [35]. Platelets may play a key role in the induction of lung injury [36]. In this study, the lungs tended to be more severely damaged in high PLT dose group, although not reaching statistical significance. Although an optimal PLT-to-RBC ratio of 1.6 has been suggested [16], however, it remains to be determined whether higher doses of PLTs in massively transfused patients increases the risk of lung injury. Other organ failures were not different between groups, suggesting that a high PLT dose does not augment the risk of organ injury. Another explanation may be that the experiment may not have lasted long enough to detect differences in organ failure. Alternatively, animals received equal amounts of transfusion volume, which may have resulted in equal organ failure. Interleukin-6 and interleukin-8 were unmeasurably low after 6 hours, which could be explained by the earlier described pattern in cytokine production during trauma and hemorrhage, in which IL-6 dropped below the detection limit [37].

Platelets actively secrete HMGB-1 [17], which may contribute to thrombosis [38]. However, in this study, we did not find any signs of thromboembolic events, regardless of the dose of platelets.

Also, in trauma, damaged cells release HMGB-1, which acts as trauma alarmin and is a late mediator of inflammation. In observational studies, high levels of HMGB-1 correlated with post-trauma complications, including MODS [17, 18, 38, 39]. Therefore, higher PLT doses could potentially be harmful to organs by HMGB-1-induced inflammation. However, in this study, HMGB-1 levels were elevated after trauma, without a difference between both resuscitation groups.

Our model has several limitations. First, although this model resembles the outcome of severe multiple trauma in real life, it hampers reaching statistical significance due to small numbers as well as to mortality in our control group. Second, this rat model was characterized by deranged coagulation; however, there are differences in rat ROTEM measures compared to humans [25]. Also, rat platelet count is higher than in humans [40]. Thereby, whether these results are also found in humans remains to be determined [41].

## Conclusion

In conclusion, in a rat model of trauma and polytransfusion, a high PLT dose was more effective than a standard PLT dose in treating TIC, with a trend towards reduced transfusion volumes. Also, transfusion of a high PLT dose did not aggravate organ damage. Whether a higher PLT dose may be a feasible treatment option to correct TIC needs further exploration.

## Abbreviations

ALT: Alanine transaminase
AST: Aspartate aminotransferase
BE: Base excess
CA5: Clotting amplitude at 5 min
CT: Clotting time
FiO_2_: Fractional inspired oxygen
H&E: Hematoxylin & eosin
Hb: Hemoglobin
HMGB-1: High-mobility group box 1
HR: Heart rate
MAP: Mean arterial pressure
MCF: Maximum clot firmness
MODS: Multiple organ dysfunction syndrome
PLT: Platelet
RBC: Red blood cell
TIC: Trauma-induced coagulopathy
